# Detection rate of prostate cancer following biopsy among the northern Han Chinese population: a single-center retrospective study of 1022 cases

**DOI:** 10.1186/s12957-017-1238-9

**Published:** 2017-08-29

**Authors:** Yong Jia, Lei-Yi Zhu, Yu-Xin Xian, Xiao-Qing Sun, Jian-Gang Gao, Xin-Hong Zhang, Si-Chuan Hou, Chang-Cun Zhang, Zhao-Xu Liu

**Affiliations:** 1 Department of Urology, Qilu Hospital of Shandong University, No.107, West Wen Hua Road, Jinan City, 250012 Shandong Province People’s Republic of China; 2 Department of Urology, Qingdao Municipal Hospital, Qingdao University, No.5, Middle Dong Hai Road, Qingdao City, 266071 Shandong Province People’s Republic of China; 3 Department of Endocrinology and Metabolism, The Affiliated Hospital of Qingdao University, Qingdao City, 266003 Shandong Province People’s Republic of China; 4 School of Nursing, Shandong University, Jinan City, 250012 Shandong Province People’s Republic of China

**Keywords:** Prostate cancer, Detection rate, PSA, DRE, Retrospective study

## Abstract

**Background:**

Prostate cancer is known to have ethnic and regional differences. The study aimed to clinically evaluate the detection rate of prostate cancer on transrectal ultrasonography (TRUS)-guided prostate biopsy and analyze its characteristics among the northern Han Chinese population at a single center.

**Methods:**

Between October 2009 and September 2016, a total of 1027 Chinese men, who had undergone TRUS-guided prostate biopsy at Qingdao Municipal Hospital, were retrospectively analyzed. Prostate biopsies were performed in the case of an abnormally elevated serum PSA level, and/or abnormal digital rectal examination (DRE) findings, and/or suspicious prostatic imaging findings.

**Results:**

Of the 1022 men enrolled in the analysis, 438 patients (42.8%) were diagnosed with prostate adenocarcinoma histologically. When serum PSA levels were divided into five subgroups (less than 4.0, 4.0 to 10.0, 10.0 to 20.0, 20.0 to 100.0, and ≥ 100.0 ng/ml), the detection rates of prostate cancer were 12.4, 15.9, 34.1, 66.2, and 93.8%, respectively. With serum PSA levels of 4.0 to 10.0 ng/ml, the cancer detection rates for a normal DRE and a suspect DRE finding were 13.5 and 58.2%, respectively. Accordingly, the cancer detection rates for a normal imaging and a suspect imaging finding were 13.5 and 58.2%, respectively. Besides, a large proportion of the patients were in the clinically advanced stage.

**Conclusions:**

The present study data reported a relatively higher prostate cancer detection rate of 42.8% and that the majority of the patients presented with clinically advanced prostate cancers within a local clinical urologic practice. An early detection and screening program for prostate cancer is of great need to reduce the burden from this disease among the northern Han Chinese population.

## Background

Prostate cancer has been recently considered to be the second most common cancer affecting men around the world, which has become a major global health concern [1]. The incidence and mortality rates of prostate cancer vary in different geographical regions. Previous studies have suggested the incidence rate of prostate cancer in Asian countries is much lower than that in the western developed countries, but with typically increasing incidence [2–4]. The large variance in the incidence of prostate cancer may be attributed to the interaction of genetic and environmental factors. In fact, recent data has shown that the incidence of prostate cancer is increasing remarkably in China [1, 5].

Prostate-specific antigen (PSA) has been commonly regarded as a basic and important screening marker for earlier diagnosis of prostate cancer [6, 7]. After Catalona et al. confirmed a PSA cutoff of 4 ng/ml as the threshold for conducting prostate biopsies [8], it has become the basic information to determine the necessity of performing prostate biopsy. Emerging evidence has demonstrated that it has led to a dramatic increase in the incidence of prostate cancer around the world since the introduction of PSA in 1986 [9]. Besides, digital rectal examination (DRE) and transrectal ultrasonography (TRUS) are another two common methods used to screen for signs of prostate cancer [10, 11].

Prostate cancer is known to have ethnic and regional differences. Even in the same country, the detection rate of prostate cancer is supposed to be variable in different areas with different environmental conditions. As far as we know, there is no study available on the subject of the detection rate of prostate cancer and few data are available to describe the clinical and pathological characteristics of prostate cancers detected in the local area of Qingdao city, which is a famous costal city in Northern China.

TRUS-guided prostate needle biopsy has been generally acknowledged as a standardized procedure for the definite diagnosis and staging of prostate cancer in clinical practice [11]. TRUS-guided prostate biopsy was introduced to our department in 2009, which was the first institution to perform prostate biopsy within the local area of Qingdao city. In the present study, we retrospectively examined our clinical database of prostate biopsies from 2009 to 2016 and aimed to assess the detection rate of prostate cancer on biopsy in the northern Han Chinese population according to the serum PSA level, DRE finding, prostatic imaging finding, and subject’s age.

## Methods

### Study cohort

From October 2009 to September 2016, a total of 1027 patients, who had undergone TRUS-guided prostate biopsy at the Department of Urology in Qingdao Municipal Hospital, were retrospectively investigated. All the patients in our study belonged to the Chinese Han nationality. Each patient received prostate biopsy for the first time. The main indications for prostate biopsy included an abnormally elevated serum PSA level, and/or abnormal DRE findings, and/or abnormal prostatic imaging findings (mainly including ultrasonography and MRI of the prostate).

The detailed indications for biopsy at our department were listed as follows: (1) a suspicious lesion identified by DRE, regardless of PSA level; (2) a suspicious area defined by ultrasound or MRI, irrespective of PSA level; (3) a PSA level greater than 10 ng/ml, regardless of the values of f/t PSA and PSA density; and (4) an abnormal value of f/t PSA or PSA density if the PSA level was 4.0 to 10.0 ng/ml.

PSA concentration was determined using the chemiluminescent method in our hospital (Roche Diagnostics, Mannheim, Germany). Blood samples were collected before DRE. The exact value of the PSA level could not be measured if the PSA level was greater than 100 ng/ml, which was all defined as 100 ng/ml in the data analysis. As prostate biopsy was scheduled as an inpatient procedure at our department, all the patients suspected with prostate cancer before the biopsy would routinely receive the examinations of the prostatic ultrasonography and MRI for further clinical evaluation. In addition, the patient characteristics, mainly including patient age, preoperative serum PSA concentration, and prostate volume, were collected and analyzed.

For experiments involving human subjects, the protocol was approved by the local ethics committee of Qingdao Municipal Hospital and performed in accordance with the ethical standards.

### Procedures of TRUS-guided prostate biopsy

TRUS-guided prostate biopsy was scheduled as an inpatient procedure at our department. Prostate biopsy was conducted after patients signed an informed consent. The same two urologic surgeons (Dr. Lei-Yi Zhu and Dr. Yong Jia), who were experienced with examination of prostate cancer patients and prostate sonography, performed all the DREs, the ultrasound examinations of the prostate, and the prostate biopsies at our department.

The patients were placed in the lateral decubitus position. Preoperatively, the DRE was initially performed by one of the same two urologic surgeons. Subsequently, the prostate of each patient was evaluated using an ultrasound equipment (Mindray Bio-Medical Electronics Co. Ltd., Shenzhen city, China) equipped with a 6-MHz bi-convex probe. Transverse and longitudinal section images were obtained. Prostate volumes were calculated using the prostate ellipsoid formula (volume = 0.52 × length × width × height). Then, all prostate biopsies were performed transrectally using an 18-gauge needle under ultrasound guidance after intravenous anesthesia. The 18-gauge biopsy needle was attached to a dedicated spring-loaded biopsy gun (MC1825, Bard Peripheral Vascular Inc., Tempe, AZ, USA). Each patient underwent a systemic 10-core biopsy plus an additional core from each suspicious area detected by TRUS. The obtained prostate-biopsy specimens were analyzed by pathologists at our institution. Tumor grade was assessed according to the Gleason scoring system [12]. Clinical staging of prostate cancer was evaluated according to the cancer staging manual of the American Joint Committee on Cancers [13]. Clinical variables and pathological features were recorded in our prostate cancer database.

### Perioperative management

The patients were asked to stop the antiplatelet- and anticoagulant-related drugs at least 1 week preoperatively. Besides, all the cases were asked to receive antibiotic prophylaxis with metronidazole (400 mg) three times a day orally starting a day before the procedure for 3 days. Bowel preparation of each patient was performed using a cleansing enema on the morning of the biopsy. After the TRUS-guided prostate biopsy, each patient received a course of intravenous antibiotics with a second generation cephalosporin for 3 days after the prostate biopsy.

#### Statistical analysis

All the data were analyzed using SPSS version 19.0 (SPSS Inc., Chicago, IL, USA). Continuous data are presented as mean ± SD. Categorical variables are presented as numbers and percentages. Student’s *t* test was used for the continuous data. A chi-square analysis was used for categorical variables. Differences were considered statistically significant if the *P* value was less than 0.05.

## Results

### Patient characteristics and grouping

From the 1027 men biopsied, 2 men diagnosed with urothelial cancer, 1 patient diagnosed with sarcoma, 1 patient diagnosed with lymphoma, and 1 man diagnosed with adenocarcinoma of intense primary origin histologically were excluded, leaving a total of 1022 biopsy results for further analysis. Of these patients, a total of 438 cases were diagnosed with prostate adenocarcinoma histologically in our study, which was defined as the cancer group, resulting in a detection rate of 42.8%. The remaining histological diagnoses were benign prostatic hyperplasia in 503 cases (86.1%), low-grade prostatic intraepithelial neoplasia (PIN) in 57 cases (9.8%), and high-grade PIN in 24 cases (4.1%), which were all defined as the non-cancer group for further analysis (*n* = 584).

The patient characteristics of the whole population are shown in Table 1. The mean age of the study population (*n* = 1022) was 71.28 ± 8.39 years old (age range 41 to 92 years). Further analysis showed that the mean age of the patients in the cancer group was significantly older than that in the non-cancer group (73.54 ± 7.91 vs. 69.53 ± 8.25 years, *P* < 0.001). In addition, the overall mean prostate volume was 49.32 ± 23.35 ml (ranging from 12.30 to 168.60 ml). Significant difference was also observed between the cancer group and non-cancer group (40.86 ± 21.64 vs. 58.12 ± 27.75 ml, *P* < 0.001).

**Table 1:** Baseline characteristics of the study participants

Items	Cancer group	Non-cancer group	Overall	*P* value
Patients (*n*)	438	584	1022	
Age (years)	73.54 ± 7.91	69.53 ± 8.25	71.28 ± 8.39	< 0.001
PV (ml)	40.86 ± 21.64	58.12 ± 27.75	49.32 ± 23.35	< 0.001

*PV* prostate volume

### Subgroup analysis of the prostate cancer detection rate in the cohort

Subgroup analysis of the prostate cancer detection rate was further performed according to the PSA levels, DRE findings, prostatic imaging findings, and the age criterion. When serum PSA levels were subdivided into five categories according to the serum PSA levels (less than 4.0, 4.0 to 10.0, 10.0 to 20.0, 20.0 to 100.0, and ≥ 100.0 ng/ml), the detection rates of prostate cancer were 30.0, 22.6, 36.0, 59.1, and 93.5%, respectively (Table 2).

**Table 2:** Detection rate of prostate cancer according to serum PSA level

PSA (ng/ml)	Patients (*n*)	Patients with PC (*n*)	Detection rate (%)
0–4	70	21	30.0
4–10	270	61	22.6
10–20	342	123	36.0
20–100	247	146	59.1
≥ 100	93	87	93.5
Overall	1022	438	42.8

*PSA* prostate-specific antigen, *PC* prostate cancer

With a normal DRE finding, the five groups with PSA levels of less than 4.0, 4.0 to 10.0, 10.0 to 20.0, 20.0 to 100.0, and ≥ 100.0 ng/ml had the cancer detection rates of 29.7, 13.5, 25.6, 55.6, and 0%, respectively. For patients with suspicious DRE findings, the corresponding detection rates of prostate cancer for the subgroups stratified according to PSA levels (less than 4.0, 4.0 to 10.0, 10.0 to 20.0, 20.0 to 100.0, and ≥ 100.0 ng/ml) were 33.3, 58.2, 59.6, 62.8, and 96.7%, respectively. Normal prostatic imaging findings with different serum PSA levels were divided into five subgroups: less than 4.0, 4.0 to 10.0, 10.0 to 20.0, 20.0 to 100.0, and ≥ 100.0 ng/ml. The corresponding rates of prostate cancer detection were 27.5, 11.2, 25.7, 54.4, and 0%, respectively. With suspicious prostatic imaging findings, the corresponding prostate cancer detection rates for the groups with PSA levels of less than 4.0, 4.0 to 10.0, 10.0 to 20.0, 20.0 to 100.0, and ≥ 100.0 ng/ml were 66.7, 48.2, 52.3, 61.3, and 100%, respectively (Table 3). The detection rates of prostate cancer in subjects aged 50 or less years, 51 to 60 years, 61 to 70 years, 71 to 80 years, and older than 80 years were 7.1, 28.1, 33.3, 48.7, and 62.2%, respectively (Table 4).

**Table 3:** Subgroup analysis of prostate cancer detection rate according to DRE and imaging findings in the different ranges of PSA levels

PSA (ng/ml)	Prostate cancer detection rate (%) (no./total no.)			
	Negative DRE	Positive DRE	Negative imaging	Positive imaging
0–4	29.7 (19/64)	33.3 (2/6)	27.5 (11/40)	66.7 (20/30)
4–10	13.5 (29/215)	58.2 (32/55)	11.2 (21/187)	48.2 (40/83)
10–20	25.6 (61/238)	59.6 (62/104)	25.7 (54/210)	52.3 (69/132)
20–100	55.6 (70/126)	62.8 (76/121)	54.4 (43/79)	61.3 (103/168)
≥ 100	0 (0/3)	96.7 (87/90)	0 (0/0)	100.0 (93/93)
Overall	27.7 (179/646)	68.9 (259/376)	25.0 (129/516)	64.2 (325/506)

*DRE* digital rectal examination, *PSA* prostatic-specific antigen

**Table 4:** Subgroup analysis of prostate cancer detection rate according to serum PSA level and age criterion

PSA (ng/ml)	Prostate cancer detection rate (%) (no./total no.)				
	≤ 50 years	51–60 years	61–70 years	71–80 years	> 80 years
0–4	100 (1/1)	33.3 (3/9)	21.9 (7/32)	31.8 (7/22)	50 (3/6)
4–10	0 (0/7)	10.8 (4/37)	16.7 (17/102)	28.8 (30/104)	50 (10/20)
10–20	0 (0/6)	23.1 (9/39)	30.6 (33/108)	40.1 (55/137)	50 (26/52)
20–100	0 (0/0)	55.6 (10/18)	47.7 (31/65)	61.5 (72/117)	70.2 (33/47)
≥ 100	0 (0/0)	100 (4/4)	88.5 (23/26)	95.6 (43/45)	94.4 (17/18)
Total	7.1 (1/14)	28.1(30/107)	33.3 (111/333)	48.7 (207/425)	62.2 (89/143)

*PSA* prostatic-specific antigen

### Clinical and pathological features of the detectable prostate cancers

The distribution of the detectable prostate cancers in terms of the Gleason score according to different PSA ranges is summarized in Table 5. Of all 438 detectable cancers, 107 cases (24.4%) presented with a Gleason score of less than 7, 129 with a Gleason score equal to 7 (29.5%), and 202 (46.1%) with a Gleason score greater than 7. These data showed that a major proportion of high-risk and poorly differentiated prostate cancers (Gleason scores 8 to 10) in our study tended to be prevalent in the men at the time of diagnosis in the initial round of examination. The corresponding percentages of poorly differentiated cancer in each subgroup of the PSA levels were 33.3, 32.8, 30.9, 50.0, and 73.6%, and there was a trend that the rate was greater as the PSA level increased.

**Table 5:** Distribution of the detectable prostate cancers in terms of Gleason score

PSA (ng/ml)	No. of PC	Gleason < 7	Gleason = 7	Gleason > 7
0–4	21	10 (47.6%)	4 (19.1%)	7 (33.3%)
4–10	61	22 (36.1%)	19 (31.1%)	20 (32.8%)
10–20	123	43 (35.0%)	42 (34.1%)	38 (30.9%)
20–100	146	30 (20.5%)	43 (29.5%)	73 (50.0%)
≥ 100	87	2 (2.3%)	21 (24.1%)	64 (73.6%)
Total	438	107 (24.4%)	129 (29.5%)	202 (46.1%)

*PSA* prostate-specific antigen, *PC* prostate cancer

The stratification of clinical T stage according to subgrouping of the PSA level is described in Table 6. Our data showed that there were 26 prostatic cancer patients (6.0%) in clinical stage T1, 97 (22.1%) in clinical stage T2, 139 (31.7%) in clinical stage T3, and 176 (40.2%) in clinical stage T4. Our results showed that the more clinically advanced cancers in this series were predominant in these data in the men in terms of the clinical T stage at time of diagnosis. The corresponding percentages of clinical stage T4 in each subgroup of the PSA levels were 6.0, 22.1, 31.7, 40.2, and 73.6% and were greater as the PSA level increased.

**Table 6:** Stratification of clinical T stage of the detectable prostate cancers according to subgrouping of PSA level

PSA (ng/ml)	No. of PC	T1	T2	T3	T4
0–4	21	4 (19.0%)	11 (52.4%)	6 (28.6%)	0 (0.0%)
4–10	61	2 (3.3%)	18 (29.5%)	23 (37.7%)	18 (29.5%)
10–20	123	12 (9.8%)	25 (20.3%)	38 (30.9%)	48 (39.0%)
20–100	146	8 (5.5%)	36 (24.6%)	43 (29.5%)	59 (40.4%)
≥ 100	87	0 (0.0%)	7 (8.0%)	29 (33.3%)	51 (58.7%)
Total	438	26 (6.0%)	97 (22.1%)	139 (31.7%)	176 (40.2%)

*PSA* prostate-specific antigen, *PC* prostate cancer

## Discussion

According to the National Central Cancer Registry of China, prostate cancer is the second most common urologic cancer in China [5]. The 2015 survey by the National Office for Cancer Prevention and Control of China showed that the incidence registration rate of prostate cancer was 2.4% of all cancer occurring in men and was seventh in prevalence [5]. Prostate cancer incidence in China has been rapidly increasing, likely owing to a growing elderly population, impact of an increasingly westernized lifestyle, and improvement of detection methods [5]. Evidence has suggested that different geographical regions also have varying incidence and mortality [5]. However, there is still no basic information for the prostate cancer detection rate according to the PSA level, DRE, and subject age criteria in our local area. Our clinical results showed that the overall detection rate for prostate cancer in our single-center study was 42.8%.

It has been recently reported that transperineal prostate biopsy could improve the detection rate compared with transrectal approach [14]. However, there are also reports suggesting that cancer detection rates are comparable with both approaches [15, 16]. As the transrectal approach has the advantages of simpler procedures, lower rate of complications, and lower cost, the transrectal approach is performed for most prostate biopsies in our daily clinical practice.

Ng and his colleagues demonstrated that the overall detection rate for prostate cancer was 52% in the initial prostate biopsy in one teaching-hospital urologic practice of Australia [17]. Our result was significantly lower than that in the above study. The possible reason may be due to the ethnic difference in the detection rates of prostate cancer. However, when comparing the detection rate with that of Korean men with similar ethnicity of Chinese men in the study by Yang et al. in 2006, our data showed that the cancer detection rate was higher than that in his study (42.8 vs. 39.7%) [18]. The similar result was also observed when comparing our results with that in another study of Korean men by Seo et al. in 2007 (42.8 vs. 32.7%) [19]. According to our limited knowledge, the difference may be attributed to the following possible reasons. For one thing, these two studies were both multicenter studies with larger populations, while our study was a single-center retrospective study with less patients. For another, emerging evidence has demonstrated that extensive systemic prostate biopsy with increased cores could significantly improve the detection of prostate cancer [20–22]. The patients in the above two Korean studies underwent different biopsy methods, including 6-, 8-, 10-, and 12-core biopsies, while the patients in our study all underwent a systemic 10-core biopsy plus an additional core from each suspicious area detected by TRUS.

In the present study, further interpretation showed that the cancer detection rates for PSA subgroups of 4.0 to 10.0, 10.0 to 20.0, 20.0 to 100.0, and ≥ 100.0 ng/ml were 22.6, 36.0, 59.1, and 93.5%, respectively (Table 2). These data may suggest that prostate cancer detection rate increased with PSA levels. With PSA levels between 4.0 and 10.0 ng/ml, the detection rate of prostate cancer in our study was 22.6%. This rate was also higher than that of Korean men in the recent two studies by Yang et al. and Seo et al. (15.9 and 19.6%, respectively). In the cases with a PSA level greater than 10.0 ng/ml, the detection rate for our study was 52.2%, which was similar to the 53.7% rate for the Korean men in the study by Seo et al. [19].

As is widely known, the most effective method to increase the detection rate of prostate biopsy is to take the DRE findings and serum PSA level into account concomitantly. However, DRE is a subjective test dependent on the examiner. In order to decrease the subjectivity and bias of evaluating the DRE, all patients were examined by the same two urologists undertaking biopsy before the procedure in our study. Accumulating evidence has shown that the detection rate of prostate cancer with suspicious DRE findings was obviously higher than that with normal DRE findings among different PSA subgroups [17–19]. Similar to the above results, it was also found in our study that significant differences in the detection rate were found according to the DRE findings in the PSA range of 4.0 to 10.0 ng/ml and 10.0 or more to less than 20.0 ng/ml (Table 3). Besides, we also found that the detection rate of prostate cancer with suspicious prostatic imaging findings was higher when compared with that with normal findings in the PSA subgroups of 4.0 to 10.0 and 10.0 to 20.0 ng/ml (Table 3). What is more, evidence has reported that serum PSA levels in cancer-free men were correlated directly with age [23]. It is also acknowledged that aging increases the risk of prostate cancer. In our present study, the detection rates of prostate cancer in patients aged 50 or less years, 51 to 60 years, 61 to 70 years, 71 to 80 years, and older than 80 years were 7.1, 28.1, 33.3, 48.7, and 62.2%, respectively, indicating that the detection rate of prostate cancer also increased according to age (Table 4).

The clinical and pathological features of prostate cancers detected in our study were further analyzed. It was found that a major proportion of high-risk and poorly differentiated prostate cancers in our study tended to be prevalent in the men at the time of diagnosis in the initial biopsy (Table 5). Regarding the clinical T stage, it should be noted that more clinically advanced cancers were predominant in these data (Table 6). As a result, the proportion of advanced disease seemed very high compared with that in western countries. The possible reason may be due to that this series is not drawn from a screening population of men. As a result, the majority of the total patients came to the urologic department because of different urologic symptoms for medical care. Practically, about 33% of the men in our study had a PSA level of greater than 20 ng/ml. Because of the high morbidity and mortality rate of prostate cancer, several international early detection and screening studies have been initiated to investigate the effect on prostate cancer mortality [24–26]. One recent 13-year follow-up outcome of the European Randomized Study of Screening for Prostate Cancer (ERSPC) has demonstrated a substantial reduction in prostate cancer mortality attributable to early detection and screening [27]. Therefore, it is of great need to conduct the early detection and screening programs of our local area to reduce the burden caused by prostate cancer, as most of the prostate cancer patients were advanced in clinical staging and pathological grade.

There were some limitations of our study, of which we were aware. For one thing, the study was limited by the nature of the retrospective study. For another, our study was also limited by the small number of patients and the single-center study. Thus, a study of a large population and multiple centers is of great need for a better clinical understanding of prostate cancer. In spite of these caveats, our present data would represent the detection rate of prostate cancer on biopsies according to the serum PSA level, DRE findings, and imaging findings in an actual practical setting for northern Han Chinese men.

## Conclusions

In the present study, our data showed that the overall prostate cancer detection rate in our single-center study was 42.8%. The detection rate of prostate cancer with a PSA level of 4.0 to 10.0 ng/ml was 22.6%. With serum PSA levels of 4.0 to 10.0 ng/ml, the cancer detection rates for patients with normal DRE and suspicious DRE were 13.5 and 58.2%, respectively, while those for the patients with normal imaging finding and suspicious imaging finding were 11.2 and 48.2%, respectively. Besides, the majority of the patients presented with clinically advanced cancers. These data may reflect the regional profile and current situation of prostate cancer among the northern Han Chinese population in our local area, providing useful information in developing specific policies and programs for better cancer control and reducing the burden and suffering caused by prostate cancer.

## Abbreviations

DRE: Digital rectal examination
PIN: Prostatic intraepithelial neoplasia
PSA: Prostate-specific antigen
TRUS: Transrectal ultrasonography
